# Comparison of Time to Pregnancy in *In Vitro* Fertilisation between Endometriosis and Nonendometriosis

**DOI:** 10.1155/2024/4139821

**Published:** 2024-09-06

**Authors:** Hartanto Bayuaji, Artha Falentin Putri Susilo, Kevin Dominique Tjandraprawira

**Affiliations:** ^1^ Department of Obstetrics and Gynecology, Faculty of Medicine Universitas Padjadjaran Dr. Hasan Sadikin General Hospital, Bandung, Indonesia; ^2^ Bandung Fertility Center Limijati Women and Children Hospital, Bandung, Indonesia

## Abstract

**Background:**

This study is to compare the time to pregnancy (TTP) between patients with endometriosis and nonendometriosis undergoing *in vitro* fertilisation (IVF).*Material and Methods.*This is an observational retrospective cohort study. We included 291 patients (53 with endometriosis and 238 without endometriosis) achieving biochemical pregnancy, whether singleton or multifetal (serum beta-hCG >5 mIU/mL), between 1st January 2014 and 31st March 2020. We excluded patients with incomplete case notes and those declining participation. Time to pregnancy is the interval between the time when infertility was established to the date of confirmed biochemical pregnancy, expressed in months. Endometriosis diagnosis includes any form of endometriosis through surgical confirmation. A statistical analysis was done through the Mann–Whitney *U* test. Time to pregnancy was assessed through the Kaplan–Meier test. A *p* value <0.05 is considered statistically significant.

**Results:**

Endometriosis patients had a shorter infertility duration (4 years vs. 5 years, *p*=0.024). Both groups had similar median age and body mass index at presentation. There was no significant difference in the TTP between endometriosis and nonendometriosis groups (57.7 vs. 70.9 months, *p*=0.060), further confirmed by a Cox regression test incorporating confounders (IVF protocol (OR: 1.482, 95% CI 0.667–3.292, and *p*=0.334) and type of the cycle (OR 1.071, 95% CI 0.803–1.430, and *p*=0.640)). The endometriosis group reached the maximum cumulative pregnancy rate at around 169 months postinfertility diagnosis, whilst the nonendometriosis group at around 255 months postinfertility diagnosis.

**Conclusion:**

Time to pregnancy between endometriosis and nonendometriosis is not significantly different. However, infertility among patients with endometriosis tends to be shorter.

## 1. Background

Infertility is the failure to conceive after 12 months of regular and unprotected sexual intercourse [1]. Whilst infertility may be contributed by female and/or male pathologies, endometriosis is a significant cause of female infertility [2]. Data vary but the monthly fecundity rate (MFR) is significantly reduced among women with endometriosis (2–10%) compared to healthy cohorts (15–20%) [2].

Endometriosis is a chronic disorder due to ectopic endometrium that responds to the monthly hormonal cycle [3]. Its main symptom is chronic cyclic pain that may manifest to chronic continuous pelvic pain [3]. Patient complaints may vary, including complaints of bowel movement, complaints of urination, dyspareunia, and infertility. [3].

Endometriosis is often diagnosed late with a significant lag time between the first appearance of symptoms and definitive diagnosis. Even in developed countries, patients may remain undiagnosed for up to 7 years [4]. Understandably, infertile patients with endometriosis present themselves at an advanced stage, having experienced a longer and more severe preoperative symptom and higher healthcare utilization costs [5]. Surgery is thus necessary, with patients often recommended to undergo IVF subsequently [6].

Endometriosis is related to infertility through various mechanisms. The presence of ectopic endometrium anywhere in the peritoneal cavity incites slow yet progressive damage. Its continuous low-grade inflammation in the reproductive tract impairs folliculogenesis, fertilisation, embryo transport, and subsequent implantation. The inflammation also causes significant pelvic adhesions distorting normal pelvic anatomy, dyspareunia reducing the frequency of sexual intercourse, ovarian damage due to the endometriosis itself and/or endometrioma and its subsequent corrective surgeries, and prolonged anovulation due to medical treatments. A combination of one or more mechanisms mentioned contributes to infertility [6].

The link between endometriosis and infertility leads to a significant increase in assisted reproductive technology (ART) utilization particularly *in vitro* fertilisation (IVF). Multiple factors, in addition to the underlying pathology, influence IVF procedures success rates [7]. A crucial parameter often overlooked is the time to pregnancy (TTP), which is how long patients recognise their disease, opt for consultation, enrol in an IVF program, and eventually conceive [8]. This is pertinent for endometriosis as patients often require years to establish a diagnosis, cited as the delay in endometriosis management [9]. There are still limited data on the TTP in IVF amongst patients with endometriosis compared to nonendometriosis patients, including those in Indonesia. This study was performed to compare the time to pregnancy in IVF between endometriosis and nonendometriosis patients to further guide clinical management accordingly.

## 2. Materials and Methods

### 2.1. Study Design and Setting

This is an observational retrospective cohort study recruiting all patients achieving biochemical pregnancy, whether singleton or multifetal, defined as serum human chorionic gonadotropin hormone (beta-hCG) level >5 mIU/mL, at two tertiary fertility centres in Bandung, Indonesia, between 1st January 2014 and 31st March 2020. The exclusion criteria included patients with incomplete case notes and those declining to participate when contacted regarding their incomplete patient follow-up data. This study is reported according to the Strengthening the Reporting of Observational Studies in Epidemiology (STROBE) guideline. [10].

### 2.2. Variables

Patients with endometriosis were those diagnosed with any form of endometriosis (superficial endometriosis, deep infiltrating endometriosis, and endometrioma) through surgical confirmation. Patients without endometriosis were those diagnosed with other aetiologies of infertility, including tubal factor infertility, ovulatory disorders, unexplained infertility, and male-factor infertility. Among patients without endometriosis, some would have had multiple aetiologies. There should be a patient with endometriosis and another form of infertility (e.g., tubal factor infertility or ovulatory disorder), they would be classified into the endometriosis group. This decision is made by a consensus of all three authors (HB, AFPS, and KDT).

### 2.3. Participants

Upon consulting the patient register at the fertility centre, each patient case notes were consulted for the outcome of patients' programs. If patients moved out of town or opted to deliver in a separate healthcare facility, we would contact the patients regarding their subsequent outcomes. They should decline to share such information, and their participation was excluded. We collected the following data: age at marriage, duration of marriage, age at presentation, body mass index (BMI) at presentation, duration of infertility, and TTP. Duration of marriage was self-reported, defined as the time between the age at presentation and the age at marriage. Duration of infertility was defined as the duration of marriage deducted by 1 year. Time to pregnancy was defined as the interval between the time when infertility was established (infertility diagnosis) to the date of confirmed pregnancy, expressed in months, as described by Ziller et al. [8]. For this study, we defined a confirmed pregnancy through a biochemical pregnancy with a beta-hCG level >5 mIU/mL. The measurement of the beta-hCG level at 2 weeks after embryo transfer is a local protocol at our facilities.

### 2.4. Statistical Methods

A customised spreadsheet is used to tabulate all patient data on Microsoft Excel for Mac version 16.16.3 (Microsoft, Redmond, USA) and the analysis performed on Statistical Product and Service Solutions (SPSS) software version 25 for Mac (IBM Corp, Armonk, New York, USA). Descriptive statistics are performed as appropriate. Analytical statistics are performed using the *t*-test or Mann–Whitney *U* test as required. The Kaplan–Meier analysis with the log-rank test is performed to analyse the point-to-point estimates on the TTP between endometriosis and nonendometriosis patients. A Cox regression test was performed to analyse the influence of covariates on the results. A *p* value <0.05 is considered statistically significant. The power of the study is set at 80%.

The ethical approval of this study was granted by the Health Research Ethics Committee of Universitas Padjadjaran under the following registration number: LB.02.01/X.6.5/98/2022.

## 3. Results

**Figure 1:**
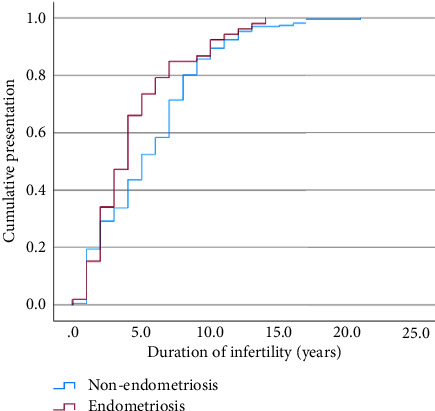
The Kaplan–Meier curve of duration of infertility plotted against patient's cumulative presentation to infertility clinic (made on Statistical Product and Service Solutions (SPSS) software version 29 for Mac (IBM Corp, Armonk, New York, USA)).

We recruited 363 patients over a 6-year-period at our centre. However, 72 patients (72/363, 19.8%) had to be excluded due to incomplete patient case notes and the patients' refusal to participate. Our final population involved 291 patients of which 53 had endometriosis and 238 did not (Table 1). The data were not normally distributed. Both groups had similar average age and BMI at presentation to our clinics. There was a significant difference in the patients' durations of marriage (years), hence their durations of infertility (years). Endometriosis patients tended to have a shorter median duration of infertility before they sought IVF (4 years vs. 5 years, *p*=0.049) (Figure 1).

**Figure 2:**
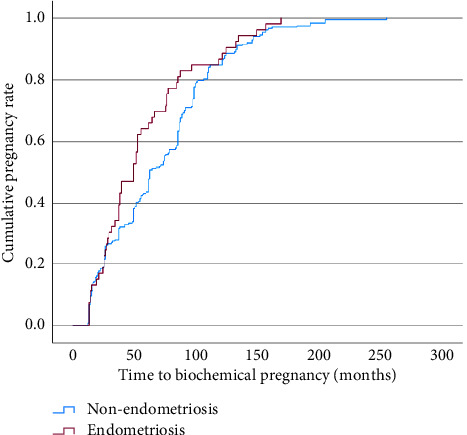
The Kaplan-Meier curve of time to pregnancy between endometriosis and non-endometriosis patients (made on Statistical Product and Service Solutions (SPSS) software version 29 for Mac (IBM Corp, Armonk, New York, USA)).

**Figure 3:**
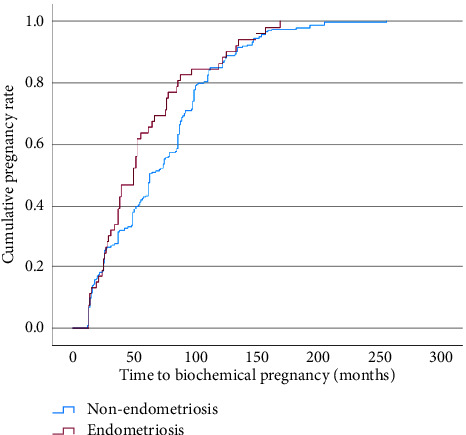
The Cox regression test on the cumulative pregnancy rate between endometriosis and nonendometriosis patients after accounting for confounding factors (IVF protocol and IVF cycle type) (made on Statistical Product and Service Solutions (SPSS) software version 29 for Mac (IBM Corp, Armonk, New York, USA)).

There was no significant difference in the average TTP between the endometriosis and nonendometriosis groups (57.7 months vs. 70.9 months, *p*=0.060), respectively . The Kaplan–Meier curve shows a nonsignificant difference of the cumulative pregnancy rate between endometriosis and nonendometriosis patients, evident from a log-rank test (*p*=0.058) (Figure 2). We performed a Cox regression test to measure the impact of the confounding factors, namely, the type of IVF protocol employed (OR: 1.482, 95% CI 0.667–3.292, *p*=0.334) and the type of cycle (frozen vs. fresh) (OR 1.071, 95% CI 0.803–1.430, *p*=0.640). We found that they did not significantly influence our findings, in which the time to pregnancy was still not significantly different between endometriosis and nonendometriosis patients (Figure 3).

**Figure 4:**
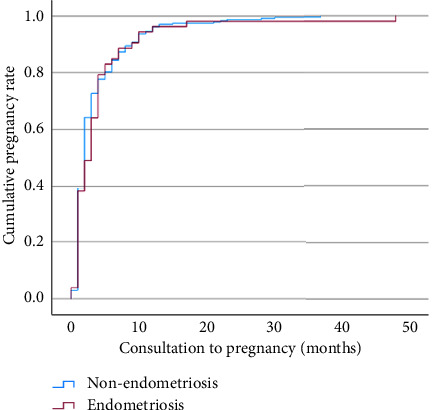
Kaplan-Meier curve of the time between consultation to pregnancy between endometriosis and nonendometriosis patients (made on Statistical Product and Service Solutions (SPSS) software version 29 for Mac (IBM Corp, Armonk, New York, USA)).

Our results show that the endometriosis group reaches themaximum cumulative pregnancy rate at around 169 months post-infertility diagnosis (>14 years) whilst the nonendometriosis group at around 255 months postinfertility diagnosis (>20 years). When we analysed the time between the patient's first consultation and their pregnancy, the endometriosis reaches the maximum cumulative pregnancy rate at around 48 months postdiagnosis whilst the nonendometriosis group at 37 months (Figure 4).

Based on our average TTP, we performed a subsequent analysis in which we divided both groups to TTP ≤60 months and >60 months. We found that patients with endometriosis were significantly more likely to be pregnant at 60 months compared to nonendometriosis patients (relative risk (RR) 1.571, 95% CI 1.077–2.290, and *p*=0.009).

## 4. Discussion

Endometriosis is a common gynaecologic disorder that continues to exert significant reproductive morbidity among Asian women [11]. This study found that the TTP among endometriosis patients does not differ significantly from nonendometriosis patients. This is surprising as one would expect endometriosis patients to achieve pregnancy in a longer time compared to those without. However, Figure 1 shows that those with endometriosis would reach the similar cumulative pregnancy rate at about 11 months later than those without endometriosis (48 months vs. 37 months).

Our findings of no significant differences in TTP for endometriosis and nonendometriosis patients may be explained in the following ways. First, our facilities are private fertility centres operating outside the national health insurance coverage and IVF is currently not covered by the Indonesian national health insurance scheme. As a result, there is an inherent selection bias for all Indonesian studies as presenting patients come from a middle to upper social class with good educational background. Our patients often already have a good prior knowledge of their diseases and tend to have had previous medical treatment and/or surgeries at other facilities prior to presenting to our facility.

Second, we did not account previous treatment histories in this analysis. Patients presenting to our facility may have had other treatments elsewhere. This is unsurprising for endometriosis patients, as their chronic pain often led them having sought treatment elsewhere. We did not include this potential confounder as the fragmented nature of the Indonesian health system which made it very difficult for us to confirm the patient's treatments and/or surgical history. Patient notes from other hospitals would be very difficult to retrieve. There may also be a significant recall bias when patients are asked to recall information on details of their surgeries and/or treatments.

Third, the patients' past endometriosis treatment would have corrected most of the pathology responsible for the failure of conception [12]. Prefumo and Rossi produced a review in which they reported that the removal of stages I and II endometriosis by laparoscopy would improve the implantation rate, pregnancy rate, and live birth rate following ART [6]. This also applies to endometriomas [6]. This may have allowed the patient to gain an advantage in IVF compared to patients without endometriosis. This might be the explanation behind the earlier TTP of patients with endometriosis, even after being stratified by age.

We also found that those with endometriosis often presented earlier to the fertility centre compared to those without and they tended to present after 4 years of infertility. This is in line with another study by Rowe et al. reporting similar findings [12]. This might be explained by the painful nature of endometriosis, which might be severe and/or debilitating among some patients. The chronic pain would prompt the patients to seek earlier treatment to alleviate their pain, which then translates to an earlier consultation regarding their infertility once the cause of their pains was recognised.

The earlier presentation time of endometriosis patients to infertility service is also noteworthy as often there is already a diagnostic delay in endometriosis which may cause women to be debilitated by chronic pain and other chronic complaints prior to being diagnosed properly [13, 14].

Another possible explanation relates to the private nature of IVF treatment itself. As it incurs a significant cost for the patients, patients with a less privileged economic background may postpone their treatment for months to years whilst attempting to save up enough funds to cover the costs of at least a single IVF cycle. This may apply to either or both groups, introducing another bias for Indonesian studies, possibly contributing to increase the lengths of infertility the patients endure. The combination of the factors above is the likely explanation.

We also found that the age did not significantly alter the TTP between those with and without endometriosis. Again, this might have been brought by the combination of factors having been described previously. In addition, for those ≤30 years old, most patients would have a good prognosis and good ovarian reserve, which would positively influence the chances of pregnancy through IVF [8].

Patients with endometriosis would perform more poorly in IVF [15, 16]. Even after surgical correction, they found that they still performed more poorly [15]. This is evident as our data show that after presenting to our facility, those with endometriosis would take a longer time to get pregnant compared to those without, with the former's maximum cumulative pregnancy rate achieved at 48 months, whilst the latter at 37 months, almost 1 year later. The reason behind such disappointing finding would be the anatomical abnormalities produced by endometriosis and the likelihood that they do not disappear even after undergoing surgeries. However, after dividing the TTP at 60 months, as expected those with endometriosis were significantly more likely to conceive than nonendometriosis patients (relative risk 1.571, 95% CI 1.077–2.290, and *p*=0.009). This is an important component for patient education in counselling their prospects of conception.

There are several limitations associated with this study: (1) under the current Indonesian national health insurance, IVF treatments are not covered. As such, those with access are often more affluent than the general Indonesian population. This is a source of selection bias. Their affluence leads to their having a high educational background with at least high school education. Thus, our population does not necessarily reflect the Indonesian infertile population in general; (2) nonendometriosis patients are comprised of a variety of disorders (for example, ovulatory disorder, tubal factor infertility, male-factor infertility, and unexplained infertility) and they affect TTP in various ways. Unfortunately, performing a separate analysis on TTP based on each type of disorder would compromise the power of the study and was not possible for us. (3) Whilst our cohort remains the biggest in our province, our patient population pales in comparison to other international studies. (4) A retrospective design like ours was not the optimum way of conducting a study for this research question. Unfortunately, time and resource constraints hindered us from performing a prospective cohort study. We agree that a multicentre, prospective cohort study would be more appropriate to answer the questions. (5) The possibility of undiagnosed endometriosis is present in the nonendometriosis group. A customised and rigorous study protocol for a future prospective study would be required to minimise this likelihood.

## 5. Conclusion

Patients with endometriosis did not differ significantly from those without endometriosis in their time to pregnancy with IVF.

## Figures and Tables

**Table 1:** Patient characteristics of endometriosis and nonendometriosis patients.

Variables (mean and standard deviation)	Endometriosis (*n*: 53)	Nonendometriosis (*n*: 238)	*p* value
Age at marriage (years)	27.0 ± 3.7	25.6 ± 4.0	0.023^∗^
Duration of marriage (years)	5.4 ± 3.4	6.8 ± 4.0	0.018^∗^
Age at presentation (years)	32.2 ± 3.8	32.1 ± 4.3	0.977^∗^
BMI at presentation (kg/m^2^)	22.4 ± 3.0	23.2 ± 3.7	0.19^∗^
Duration of infertility (years)	4.45 ± 3.36	5.62 ± 3.88	0.024^∗^
Overall time to pregnancy (months)	57.7 ± 40.9	70.9 ± 46.4	0.060^∗^

*Note.* ^∗^Mann–Whitney *U* test.

## Data Availability

Anonymised data are available upon reasonable written request to the corresponding author.

## Additional Points

*Declaration of Figures Authenticity*. All figures submitted have been made by the authors using Statistical Product and Service Solutions (SPSS) software version 29 for Mac (IBM Corp, Armonk, New York, USA). We confirm that the images are original with no duplication and have not been previously published in whole or in part.
